# Patient and Microbial Genomic Factors Associated with Carbapenem-Resistant Klebsiella pneumoniae Extraintestinal Colonization and Infection

**DOI:** 10.1128/mSystems.00177-21

**Published:** 2021-03-16

**Authors:** Zena Lapp, Jennifer H. Han, Jenna Wiens, Ellie J. C. Goldstein, Ebbing Lautenbach, Evan S. Snitkin

**Affiliations:** a Department of Computational Medicine and Bioinformatics, University of Michigan, Ann Arbor, Michigan, USA; b GlaxoSmithKline, Rockville, Maryland, USA; c Department of Electrical Engineering and Computer Science, University of Michigan, Ann Arbor, Michigan, USA; d R. M. Alden Research Laboratory, Culver City, California, USA; e David Geffen School of Medicine, University of California, Los Angeles, Santa Monica, California, USA; f Department of Medicine (Infectious Diseases), University of Pennsylvania, Philadelphia, Pennsylvania, USA; g Department of Microbiology and Immunology, University of Michigan, Ann Arbor, Michigan, USA; h Department of Biostatistics, Epidemiology, and Informatics, Perelman School of Medicine, University of Pennsylvania, Philadelphia, Pennsylvania, USA; i Department of Internal Medicine/Division of Infectious Diseases, University of Michigan, Ann Arbor, Michigan, USA; University of Southampton

**Keywords:** infection, *Klebsiella pneumoniae*, machine learning, antibiotic resistance, genomic epidemiology, hospital infections, whole-genome sequencing

## Abstract

Carbapenem-resistant Klebsiella pneumoniae (CRKP) is a critical-priority antibiotic resistance threat that has emerged over the past several decades, spread across the globe, and accumulated resistance to last-line antibiotic agents. While CRKP infections are associated with high mortality, only a subset of patients acquiring CRKP extraintestinal colonization will develop clinical infection. Here, we sought to ascertain the relative importance of patient characteristics and CRKP genetic background in determining patient risk of infection. Machine learning models classifying colonization versus infection were built using whole-genome sequences and clinical metadata from a comprehensive set of 331 CRKP extraintestinal isolates collected across 21 long-term acute-care hospitals over the course of a year. Model performance was evaluated based on area under the receiver operating characteristic curve (AUROC) on held-out test data. We found that patient and genomic features were predictive of clinical CRKP infection to similar extents (AUROC interquartile ranges [IQRs]: patient = 0.59 to 0.68, genomic = 0.55 to 0.61, combined = 0.62 to 0.68). Patient predictors of infection included the presence of indwelling devices, kidney disease, and length of stay. Genomic predictors of infection included presence of the ICEKp10 mobile genetic element carrying the yersiniabactin iron acquisition system and disruption of an O-antigen biosynthetic gene in a sublineage of the epidemic ST258 clone. Altered O-antigen biosynthesis increased association with the respiratory tract, and subsequent ICEKp10 acquisition was associated with increased virulence. These results highlight the potential of integrated models including both patient and microbial features to provide a more holistic understanding of patient clinical trajectories and ongoing within-lineage pathogen adaptation.

**IMPORTANCE** Multidrug-resistant organisms, such as carbapenem-resistant Klebsiella pneumoniae (CRKP), colonize alarmingly large fractions of patients in regions of endemicity, but only a subset of patients develop life-threatening infections. While patient characteristics influence risk for infection, the relative contribution of microbial genetic background to patient risk remains unclear. We used machine learning to determine whether patient and/or microbial characteristics can discriminate between CRKP extraintestinal colonization and infection across multiple health care facilities and found that both patient and microbial factors were predictive. Examination of informative microbial genetic features revealed variation within the ST258 epidemic lineage that was associated with respiratory tract colonization and increased rates of infection. These findings indicate that circulating genetic variation within a highly prevalent epidemic lineage of CRKP influences patient clinical trajectories. In addition, this work supports the need for future studies examining the microbial genetic determinants of clinical outcomes in human populations, as well as epidemiologic and experimental follow-ups of identified features to discern generalizability and biological mechanisms.

## INTRODUCTION

Infections due to multidrug-resistant organisms (MDROs) lead to hundreds of thousands of deaths worldwide each year (1). Carbapenem-resistant *Enterobacterales* (CRE) are a critical-priority antibiotic resistance threat that has emerged over the past several decades, spread across the globe, and accumulated resistance to last-line antibiotic agents (2, 3). In the United States (U.S.), CRE infections are primarily caused by the sequence type (ST) 258 strain of carbapenem-resistant Klebsiella pneumoniae (CRKP), which has become endemic in regional health care networks (3–7). In this background of regional endemicity, the risk of patient exposure to CRKP is high, as evidenced by alarmingly high rates of colonization, especially in long-term care settings (7, 8). However, even among critically ill patients residing in long-term care facilities, not all colonized patients develop clinical infections that require antibiotic treatment (3, 9). Currently, our understanding of the factors that influence whether a colonized patient develops an infection is incomplete.

In addition to clinical characteristics of patients (10), the genetic background of the colonizing strain may also influence the risk of infection, as there is extensive intraspecies variation in antibiotic resistance and virulence determinants harbored by K. pneumoniae (3). To date, most studies of virulence determinants have been carried out in model systems (11, 12) or examined in human populations without considering patient characteristics or clinical context (12, 13). One recent study investigated virulence determinants in K. pneumoniae clinical isolates while controlling for patient characteristics (14). However, this was a single-site study with a focus on carbapenem-susceptible K. pneumoniae, thereby not addressing the impact of genomic variation in antibiotic-resistant lineages that circulate in global health care systems.

Here, we sought to understand the importance of both patient factors and genomic features in determining whether a patient is colonized or infected with ST258 CRKP. Importantly, we restricted our comparison to patients with extraintestinal CRKP colonization versus infection. We reasoned that this comparison would reveal the patient and microbial factors that influence risk for infection when CRKP is present in an extraintestinal site, eliminating confounding by factors associated with translocation from the gastrointestinal tract. To gain an unbiased assessment of influential patient and microbial factors in a high-risk population, we leveraged a comprehensive set of all clinical isolates and patient metadata collected from 21 long-term acute-care hospitals (LTACHs) across the U.S. over the course of a year. Machine learning models rigorously trained and tested on these data revealed that patient and microbial factors were similarly predictive of ST258 CRKP colonization versus infection, indicating that both contribute to infection risk. Moreover, examination of predictive genomic features revealed genetic variation within the epidemic ST258 lineage of CRKP that was associated with increased respiratory colonization and higher infection rates.

## RESULTS

Of 355 clinical CRKP isolates from 21 LTACHs across the U.S. (15), we classified 149 (42%) of the isolates as representing extraintestinal infection based on modified National Healthcare Safety Network (NHSN) criteria (7) (see Fig. S2 and Tables S1 to S3 in the supplemental material). The rest of the isolates were classified as representing extraintestinal colonization. Stratified by anatomic site, we classified 29/29 (100%) blood isolates as infection, 69/196 (35%) respiratory isolates as infection, and 51/130 (39%) urinary isolates as infection (Table S3). More than 90% of patient isolates were from the epidemic CRKP lineage ST258 (Table S1). Patients harboring different sequence types of CRKP showed no significant differences in infection/colonization status or anatomic site of isolation, and no substantive differences in clinical characteristics (see the supplemental material). Thus, we decided to limit our analysis to ST258 to improve our ability to discern whether genetic variation within this dominant strain is associated with infection.

10.1128/mSystems.00177-21.7 TABLE S1 Infection status and anatomic site of isolation by CRKP sequence type. Download Table S1, DOCX file, 0.01 MB. Copyright © 2021 Lapp et al. 2021 Lapp et al. https://creativecommons.org/licenses/by/4.0/ This content is distributed under the terms of the Creative Commons Attribution 4.0 International license.

### The CRKP epidemic lineage ST258 shows evidence of sublineage variation in virulence and anatomic site of isolation.

**FIG 1:**
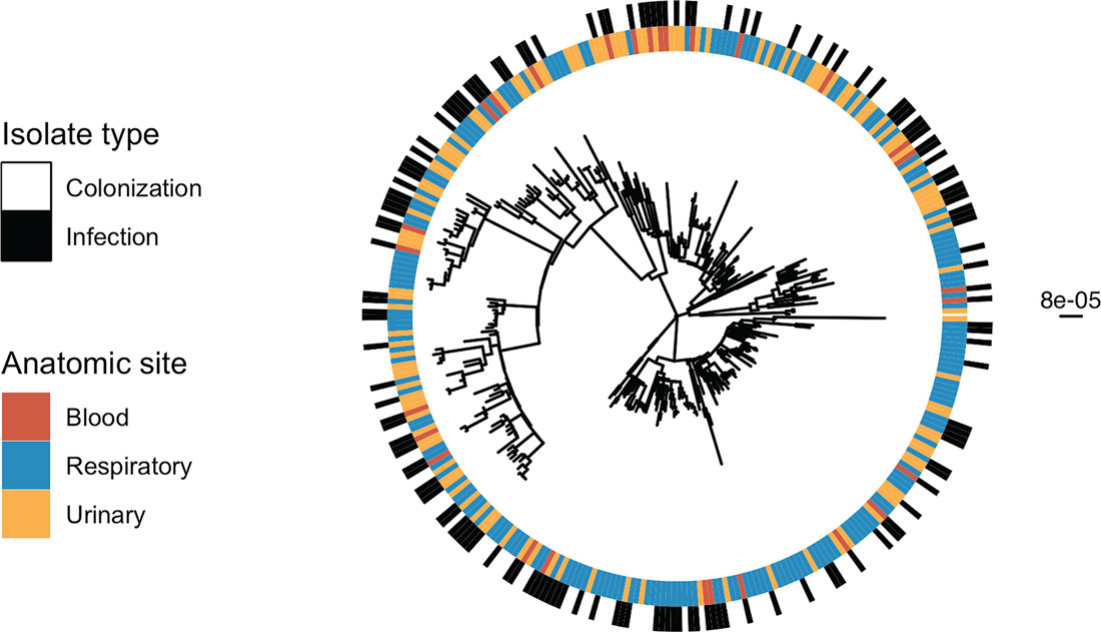
Infection and anatomic site cluster on the phylogeny. Maximum likelihood phylogenetic tree of all isolates including infection or colonization classification for each isolate and anatomic site of isolation. The scale bar to the right of the phylogeny shows the branch length in substitutions per site. Testing for nonrandom distribution of isolates on the phylogeny (see the supplemental material) revealed clustering of infection, respiratory, and urinary isolates on the phylogeny, respectively.

We next evaluated if there exist sublineages of ST258 with altered virulence properties by looking for clustering of isolates by infection on the whole-genome phylogeny (Fig. 1; also see the supplemental material) (16). Infection status was nonrandomly distributed on the phylogeny (*P* = 0.002), supporting our hypothesis that the genetic background of CRKP influences infection. We performed a similar clustering analysis to look at potential niche-specific adaptation to certain anatomic sites (Fig. 1) and found that respiratory (*P* = 0.001) and urinary (*P* = 0.013) isolates cluster on the phylogeny but blood isolates do not (*P* = 0.21). This analysis indicates that, in addition to patient features, intrastrain variation in virulence and adaptation to the urinary and respiratory tract might influence whether patients develop an infection.

### Both patient and CRKP ST258 genetic characteristics are weakly predictive of infection, with relative performance being highly facility dependent.

We next performed machine learning using L2 regularized logistic regression to quantify the ability of patient and microbial genetic characteristics to predict CRKP ST258 infection (Fig. S1). To prevent over- or underfitting and control for facility-level biases, we generated 100 train/test data splits, wherein a given LTACH was included only in either the train or test set. Each LTACH occurred a median of 24 times (range 13 to 32) in the test data split. In this way, we were able to identify patient and CRKP ST258 strain characteristics consistently associated with infection or colonization across data splits and thus across patient populations in different health care facilities.

10.1128/mSystems.00177-21.2 FIG S1 Methods overview. (A) Isolate subsets, based on anatomic site, used for different machine learning analyses. (B) Outcome used for all machine learning analyses. (C) Feature sets used for machine learning analyses (see Materials and Methods for more details). (D) Machine learning analysis pipeline: (D1) machine learning pipeline, (D2) median AUROC calculated from machine learning pipeline, and (D3) mean difference in test versus permuted AUROC for each feature (see Materials and Methods for more details). AUROC, area under the receiver operating characteristic curve. Download FIG S1, TIF file, 32.1 MB. Copyright © 2021 Lapp et al. 2021 Lapp et al. https://creativecommons.org/licenses/by/4.0/ This content is distributed under the terms of the Creative Commons Attribution 4.0 International license.

10.1128/mSystems.00177-21.3 FIG S2 There are differences in sample size and distribution of infection and anatomic site of samples across LTACHs. Distribution of colonization and infection isolates is split by source and LTACH of origin. A to U are specific LTACHs, and the geographic location by state is in parentheses. LTACH, long-term acute-care hospital. Download FIG S2, TIF file, 17.2 MB. Copyright © 2021 Lapp et al. 2021 Lapp et al. https://creativecommons.org/licenses/by/4.0/ This content is distributed under the terms of the Creative Commons Attribution 4.0 International license.

**FIG 2:**
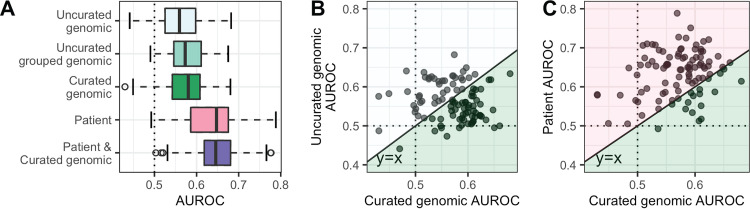
Test AUROCs for various classifiers identifying CRKP colonization versus infection vary substantially across data splits. (A) Test AUROCs for 100 L2 regularized logistic regression models different using train/test splits. All isolates from a given LTACH were included in either the training split or the testing split for each data split. We built models using five different feature sets, keeping the same 100 data splits. AUROCs of different feature sets were not significantly different. (B and C) In the right two panels, the curated genomic feature set AUROCs are compared to the uncurated genomic feature set AUROCs (B) and the patient feature set AUROCs (C). Each point is the resulting pair of AUROCs for models built with the same data split but the two respective feature sets. The dotted lines in all 3 panels indicate the AUROC for choosing an outcome randomly (0.5); anything below the line is worse than random, and anything above the line is better than random. The solid diagonal line in the right two panels is the line *y* = *x*; points below the line correspond to a higher curated genomic AUROC for that data split, and points above the line correspond to a higher uncurated genomic AUROC (B) or patient AUROC (C), respectively. The colors in panels B and C correspond to the colors in panel A; the points in a given colored area indicate that that feature set had the higher AUROC for that data split. In both cases, one feature set does not consistently outperform the other (*P* = 0.4; see the supplemental material for *P* value calculation). AUROC, area under the receiver operating characteristic curve.

First, we sought to understand if patient and genomic features were individually predictive of CRKP ST258 infection. To this end, we independently evaluated patient characteristics as well as three different genomic feature sets for their ability to classify colonization and infection. The three genomic feature sets were uncurated genomic (including single nucleotide polymorphisms [SNPs], indels, insertion sequence [IS] elements, and accessory genes), uncurated grouped genomic (variants grouped into genes, akin to a burden test, e.g., reference 17), and curated genomic (features identified using Kleborate [18]). Across the 100 different train/test splits, we observed that the average predictive performance was weak, with each of the genomic and patient feature sets predictive of infection to a similar degree (all 1st-quartile areas under the receiver operating characteristic curve [AUROCs] > 0.5; median range = 0.55 to 0.68 [Fig. 2A]; area under the precision recall curve [AUPRC] [Fig. S3A]). Additionally, no one feature set was consistently the most predictive (e.g., Fig. 2B and C; all comparisons *P* > 0.30; see the supplemental material for *P* value calculation). Furthermore, for each feature set the AUROCs were distributed such that the test AUROC ranged from below 0.5 to over 0.7, depending on how the data were split (i.e., which facilities appear in the train/test sets). This variation in model performance across different train/test sets suggests that the association of CRKP ST258 strain and patient characteristics with infection or colonization varies across facilities.

10.1128/mSystems.00177-21.4 FIG S3 Model performance for various feature sets, body sites, and machine learning methods. Model performance for 100 train/test splits used to build classifiers with L2 regularized logistic regression. (A, C, and D) Test AUPRCs used to build classifiers with L2 regularized logistic regression identifying CRKP colonization versus infection for the overall model with various classifiers. (B) Test AUROC of ST258 machine learning models for various methods. The patient and genomic feature set was used for model comparisons. (C and D) Test AUPRCs (C) and test AUROCs (D) used to build classifiers with L2 regularized logistic regression identifying CRKP colonization versus infection for respiratory and urinary models; the curated genomic feature set was used here. The dotted lines in all panels indicate the model performance for choosing an outcome randomly; anything below the line is worse than random, and anything above the line is better than random. We found that AUROC (C; also Fig. 2A) and AUPRC (A and D) both indicate that the models are slightly predictive of colonization versus infection. AUROC, area under the receiver operating characteristic curve; AUPRC, area under the precision recall curve. Download FIG S3, TIF file, 10.3 MB. Copyright © 2021 Lapp et al. 2021 Lapp et al. https://creativecommons.org/licenses/by/4.0/ This content is distributed under the terms of the Creative Commons Attribution 4.0 International license.

### Integration of patient and CRKP strain features does not improve discriminative performance of overall or anatomic site-specific models.

To determine if the predictive power of patient and genomic features is additive, and if combining these disparate feature sets improved validation on held-out facilities, we built models including both patient and curated genomic features. The discriminative performance of the models based on the combined feature set was not significantly greater than that of the individual feature sets (Fig. 2A, all *P* ≥ 0.20). Thus, despite variation in the predictive capacity of genomic and patient features across facilities (Fig. 2C), combining the two sets did not improve overall performance. Furthermore, we found that there was no significant difference in model performance between L2 regularized logistic regression, elastic net, random forest, and support vector machines with a radial basis kernel (Fig. S3B, all *P* > 0.1). Focusing on anatomic site-specific L2 regularized logistic regression models revealed similar trends, where classification performances were similar for respiratory and urinary tract-specific models, and the relative predictive capacity of patient and CRKP ST258 strain features varied across facility subsets (Fig. S3C and D).

### Some patient and genomic features consistently discriminate colonization and infection.

**FIG 3:**
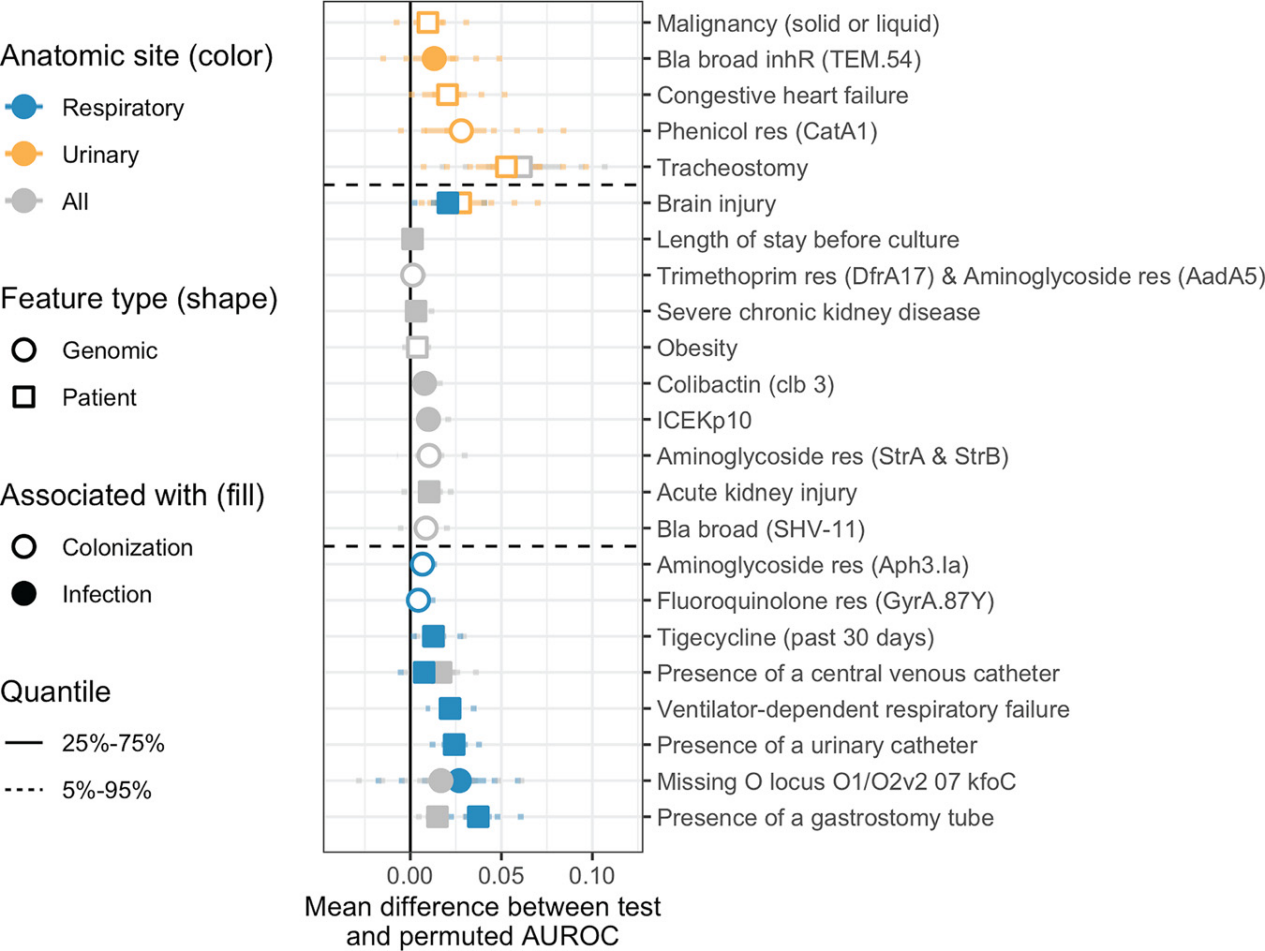
Features consistently associated with colonization or infection sometimes differ between the overall, respiratory, and urinary models. Feature-specific improvement in model performance, measured as the mean difference between test and permuted AUROC (see Materials and Methods), of features found to be consistently associated with colonization or infection in at least one of the following analyses: overall, respiratory specific, and urinary specific. We consider features to be associated with infection/colonization if the AUROC difference was greater than zero in over 75% of the 100 data splits. The vertical solid black line indicates a difference of zero (i.e., the feature provides no improvement to model performance). Horizontal dotted lines separate features associated with urinary but not respiratory isolates (top), both urinary and respiratory (or all) isolates (middle), or respiratory but not urinary isolates (bottom). Bla, beta-lactamase; res, confers resistance to that antibiotic class; AUROC, area under the receiver operating characteristic curve.

After evaluating the predictive capacity of models, we next sought to identify patient and CRKP ST258 strain characteristics that are most associated with infection or colonization. To this end, we identified those patient and genomic features that consistently improved model performance across the 100 different data splits (see Materials and Methods). Evaluating the importance of features in this way provides insight into those characteristics that generalize across different facility subsets. This approach was taken for both overall and anatomic site-specific models to identify features predictive of different anatomic sites of infection (Fig. 3 and Fig. S4).

10.1128/mSystems.00177-21.5 FIG S4 Differences in test versus mean permuted AUROC across 100 data splits for each feature. (A) Overall machine learning analysis. (B) Respiratory machine learning analysis. (C) Urinary machine learning analysis. All features included in the model are shown. We consider features to be associated with infection/colonization if the AUROC difference is greater than zero in over 75% of the 100 data splits (see Materials and Methods for more details). Features above the dotted line fall into this category. The solid gray line indicates a difference of zero (i.e., the feature provides no improvement to model performance). Bla, beta-lactamase; res, confers resistance to that antibiotic class. Download FIG S4, TIF file, 85.8 MB. Copyright © 2021 Lapp et al. 2021 Lapp et al. https://creativecommons.org/licenses/by/4.0/ This content is distributed under the terms of the Creative Commons Attribution 4.0 International license.

Several patient features were consistently associated with infection in the overall analysis, including presence of a gastrostomy tube, presence of a central venous catheter, acute kidney injury, and severe chronic kidney disease (Fig. 3), all markers of critically ill patients. Only a small number of genomic features were consistently associated with infection or colonization (Fig. 3). The genomic features associated with colonization were all antibiotic resistance determinants. Conversely, all but one of the genomic features positively associated with infection (3/4) are related to virulence. The ICEKp10 element is positively associated with infection and carries colibactin and two different types of yersiniabactin, a previously identified K. pneumoniae virulence determinant (12). Colibactin is a toxin (3), and yersiniabactin is an iron-scavenging system that has been identified in previous animal and human studies as being associated with virulence (3, 11). Additionally, insertion sequence-mediated disruption of the O-antigen biosynthetic gene *kfoC* (see Materials and Methods and Fig. S5A for insertion sequence identification) was associated with respiratory infection. The O-antigen of lipopolysaccharide (LPS) is a known antigenic marker, although association with a specific anatomic site has not been noted (19).

10.1128/mSystems.00177-21.6 FIG S5 *kfoC* contains an IS element in a subset of ST258 isolates from different LTACHs, mainly confined to one ST258 clade. (A) Phylogeny of isolates indicating the LTACH the patient was in when the isolate was obtained. Isolates from several LTACHs are present in the disrupted *kfoC* lineage or the ybt0 lineage, and isolates from these LTACHs are not confined to those lineages. The heatmap beside the ST258 phylogeny indicates information about the isolate: left to right, the O locus type identified by Kleborate (i.e., the curated genomic data), if Kleborate identified *kfoC* as missing, blast results for *kfoC* against the genome assemblies (split between 2 assemblies or entirely absent; white indicates that *kfoC* was intact), and IS elements identified in *kfoC* (white indicates no IS element detected). The scale bar to the top left of the phylogeny shows the branch length in substitutions per site. (B) Phylogeny of California LTACH isolates (US-CA) in the context of public isolates from various locations (EUR, Europe; SA, South America; US-MW, U.S. Midwest; US-NE, U.S. Northeast; US-S, U.S. South; US-W, U.S. West). Isolate location, as well as ybt0 presence and *kfoC* absence, are displayed for each sample. The scale bar to the right of the phylogeny shows the branch length in substitutions per site. Download FIG S5, TIF file, 41.2 MB. Copyright © 2021 Lapp et al. 2021 Lapp et al. https://creativecommons.org/licenses/by/4.0/ This content is distributed under the terms of the Creative Commons Attribution 4.0 International license.

### A sublineage of ST258 clade II appears to have sequentially evolved enhanced adaptation for the respiratory tract and increased virulence.

**FIG 4:**
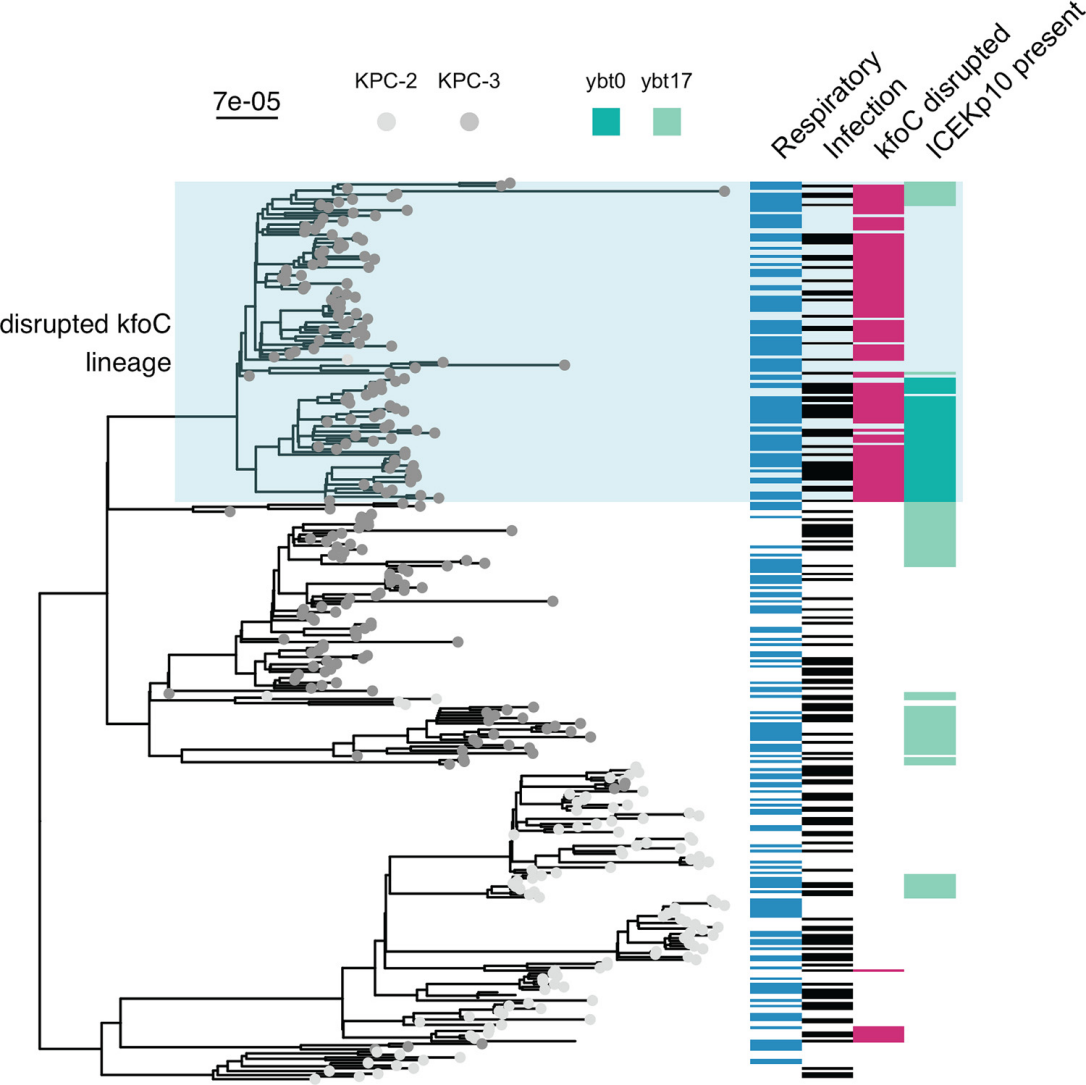
Select epidemiologic and genomic features visualized on the phylogeny indicate that a subclade of ST258 clade II may exhibit enhanced niche-specific adaptation and virulence. ST258 maximum likelihood phylogeny with the tip labels colored by KPC gene. The blue box indicates the sublineage with apparent altered niche-specific adaptation that acquires an additional virulence locus. The heatmap beside the tree indicates information about the isolate: from left to right, if it is a respiratory isolate, if it is an infection isolate, if *kfoC* is disrupted, and if it contains ICEKp10. Disrupted *kfoC* was associated with infection in the overall and respiratory machine learning analyses, and ICEKp10 presence was associated with infection in the overall analysis. The scale bar to the top left of the phylogeny shows the branch length in substitutions per site. ybt, yersiniabactin; ybt0 and ybt17 are two ybt STs defined by Kleborate.

We noted that *kfoC* disruption is largely confined to a sublineage of ST258 present across 12 LTACHs in California (Fig. 4 and Fig. S5). Consistent with this feature being associated with respiratory infection, the disrupted *kfoC* lineage is enriched in respiratory isolates (82/118 [69%] isolates in the disrupted *kfoC* lineage are respiratory isolates versus 101/213 [47%] in all other isolates; Fisher’s exact *P* = 0.0001), suggesting that this lineage is associated with increased capacity for respiratory colonization. Furthermore, a subset of isolates in the disrupted *kfoC* sublineage harbor the ICEKp10 element containing yersiniabactin. Examination of these genetic events in the context of the whole-genome phylogeny revealed that disruption of *kfoC* occurred first, followed by at least two different acquisitions of ICEKp10 (Fig. 4). Within the disrupted *kfoC* lineage, isolates with ICEKp10 are enriched in infection (31/55 [56%] isolates with ICEKp10 are infection isolates versus 16/63 [25%] isolates without ICEKp10, Fisher’s exact *P* = 0.00065), supporting an increase in virulence after acquisition of ICEKp10. It is important to note that the observed clinical associations with ICEKp10 and *kfoC* disruption do not demonstrate causality, as we cannot rule out the role of correlated genetic variation.

## DISCUSSION

There have been numerous studies aimed at identifying risk factors for health care-associated infections caused by prominent antibiotic resistance threats. For the most part, these studies have found the dominant risk factors to be linked to the magnitude of exposure (e.g., length of stay or colonization pressure), use of antibiotics, and overall comorbidity (20). What remains unclear is if in addition to these clinical features, the genetic variation in circulating resistant lineages also contributes to patient risk of infection. Here, we addressed this question for CRKP ST258 in a comprehensively sampled cohort of patients from 21 LTACHs across the U.S. Overall, we found that, while neither patient nor CRKP ST258 genetic features have high predictive accuracy on held-out test data, both feature sets were independently associated with infection, with one or the other being more predictive on different facility subsets. Moreover, the integration of clinical and genomic data led to the discovery of an emergent sublineage of the epidemic ST258 clone that may have increased adaptation for the respiratory tract and is more strongly associated with infection.

One strength of our machine learning approach is that we were able to measure the variation in discriminative performance across 100 train/test iterations that differed in which facilities were included in train and test sets. We found that performance varied greatly depending on how facilities were allocated to train and test sets, highlighting how smaller studies could overestimate or underestimate the discriminative ability of both their model and individual features. Variation in model performance across facilities could be due to facility-level heterogeneity leading to differences in the prevalence of predictive patient or genomic features in the different test sets. For instance, certain facilities may have patient populations skewed toward individuals with characteristics that are predictive of infection. Alternatively, certain geographic regions may have CRKP ST258 strains that are more virulent than in other regions. These differences could lead to a higher predictive power for certain facilities compared to others. Another possible explanation for variation in model performance is that the critically ill nature of LTACH patients may be such that most patients are actually highly susceptible to infection (i.e., many patients colonized with CRKP may ultimately develop an infection). However, it is noteworthy that despite these potential challenges in creating generalizable models, our analysis did yield predictors of infection and colonization consistent across test sets and thus across LTACHs.

We built classifiers including all genomic features as well as a curated subset of features from Kleborate (18) and found that the two are similarly predictive of infection. However, while the uncurated feature set presented challenges with downstream interpretation, our analyses on the curated genomic features (18) facilitated novel insights into potential evolutionary trajectories of anatomic site-specific adaptation and virulence. For example, we observed that disruption of the O-antigen biosynthetic gene, *kfoC*, is associated with isolation from the respiratory tract. While we cannot determine from our machine learning analysis if disruption of *kfoC* is directly causal, the biological plausibility of an altered O-antigen structure mediating evasion of innate immunity and/or other beneficial interactions with the host makes this a strong candidate for follow-up experiments. Supporting this hypothesis, a previous study found that absence of O-antigen is associated with decreased virulence, but not decreased intrapulmonary proliferation, in a murine model (21). In addition, we noted that a number of antibiotic resistance determinants were associated with colonization. We hypothesize that this observation could be a consequence of longer duration of residence being associated with increased exposure to off-target antibiotics (22). Finally, we also saw evidence that, after acquiring the virulence factors yersiniabactin and colibactin on the ICEKp10 element, the disrupted *kfoC* subclade became more strongly associated with infection, supporting the idea that circulating ST258 sublineages can evolve to become both hypervirulent and multidrug resistant (23–26).

It is important to note that the machine learning method we employed does not correct for microbial population structure. We chose this method instead of alternative bacterial genome-wide association methods because our primary interest was in quantifying the overall predictive capacity of bacterial genotype in a patient population that was collected in a comprehensive and unbiased manner (i.e., all clinical isolates from 21 facilities over 1 year). While alternative methods controlling for population structure may yield more precise estimates of the contribution of individual variants, this would obfuscate the realized contribution in our patient population and hinder direct comparison to the predictive capacity of patient features. However, this then presented the challenge of interpreting our finding that certain subpopulations of CRKP ST258 may differ in their predilection for causing infections at different sites. For instance, the *kfoC* disruption is a lineage-defining variant, and in principle other variants that define this lineage could also be causal. Here, we limited our analysis to a curated set of variants belonging to pathways known to be associated with antibiotic resistance and virulence and found that only *kfoC* disruption was associated with increased respiratory infection, thus making it a strong candidate for follow-up in *in vitro* or *in vivo* models (11). To identify novel loci whose role in human infection may not be appreciated, both computational and experimental strategies may be employed to help prioritize putative causal versus passenger variants. Computationally, investigators may search for evidence of parallelism in genotype/phenotype associations, which would bolster confidence in causality (27). Alternatively, high-throughput screens of genetic mutants in relevant model systems can help prioritize candidates. Garnering further genomic or experimental support for the direct role of a specific genetic variant would in turn increase the likelihood that those genetic markers would be predictive in new strains and patient populations.

Our study also has several important limitations related to the data available. Specifically, extraintestinal CRKP colonization versus infection for nonbloodstream isolates may be difficult to discriminate using surveillance criteria and the clinical data that were available. However, we based our definitions on established CDC criteria with modifications used previously (7). Encouragingly, we were still able to identify consistent predictors of infection, even with potential misclassifications. A second limitation is that our data set included only one clinical culture for the majority of patients, meaning that we were unable to investigate clinical or genomic features that may be associated with progression from colonization to infection. Furthermore, we do not have CRKP rectal colonization isolates and therefore cannot evaluate transition from rectal colonization to other body sites. However, we hypothesize that comparing rectal colonization to infection may be asking a subtly distinct question—namely, bacterial genetic factors that enable translocation from the gut to other body sites. In contrast, we hypothesize that our study design is ideal to identify bacterial genetic factors associated with infection once at a given body site. Additionally, we chose to focus our analysis on ST258 due to its disproportionate presence in our data set, but this makes it possible that our findings may not generalize to other sequence types. Nevertheless, ST258 is the dominant clone in the U.S., and the methods we employed here can be used to study other sequence types and other pathogens. Furthermore, our focus on the ST258 lineage led to the particularly notable finding that even within an established endemic multidrug-resistant lineage (which emerged circa 2000 [15]), there is continued evolution that influences the manifestation and outcome of infection. This highlights the importance of performing strain-specific analyses to identify continued evolution and adaptation of hospital-associated lineages. We were also limited in the patient data included in our model. It is likely that important differences in underlying patient conditions were not captured by the coarse clinical variables we included, and we also did not account for differences in genetic variation in the host (28). Other limitations include that our study was restricted to LTACH patients and had nonrandom geographic sampling. However, our restriction to LTACHs in geographic regions of endemicity has the benefit of focusing on populations at disproportionate risk for CRKP infection (8).

In conclusion, we employed a machine learning approach to quantify our ability to discriminate between CRKP colonization and infection using patient and microbial genomic features. This approach highlighted the high degree of variation in predictive accuracy across different facility subsets. Furthermore, despite modest predictive power, we identified several genomic features consistently associated with infection, indicating that variation in circulating CRKP strains contributes to infection, even in the context of the critically ill patient populations residing in LTACHs. Future work should aim to corroborate our findings with larger cohorts and follow up on strong associations to determine whether they are indeed risk factors for infection. This could ultimately help identify patients at high risk for CRKP ST258 infection and devise targeted strategies for infection prevention. Furthermore, the methods employed here can be used to study ongoing adaptation in other important MDRO lineages circulating in health care facilities.

## MATERIALS AND METHODS

### Clinical and genomic data.

We used whole-genome sequences of clinical (nonsurveillance) CRKP isolates and associated patient metadata from a prospective observational study performed in 21 LTACHs from across the U.S. over the course of a year (BioProject accession no. PRJNA415194) (15). All isolates were ordered by clinicians as part of clinical care, and clinical practice guidelines and policies are standard across sites within the network. We included only the first clinical bloodstream, respiratory, or urinary isolate from each patient (*n* = 355; see Fig. S1A in the supplemental material) and subset to only ST258 isolates for the majority of analyses (*n* = 331; Table S1; see supplemental material for reasoning). Patient metadata were obtained from electronic health records. Core genome variants were identified using a reference genome, and accessory genes were identified using Roary (29). Details about the clinical data, analysis pipeline (30), genomic data curation (15, 18, 23, 29, 31–34), and phylogenetic reconstruction (35–38) are provided in the supplemental material. While most clinical data cannot be shared, the deidentified patient ID, hospital of sample isolation, and isolation site are included in the Sequence Read Archive metadata for the BioProject.

### Outcome definition.

Our outcome of interest was colonization versus clinical infection (Fig. S1B). Based on the U.S. Centers for Disease Control and Prevention’s (CDC’s) established National Healthcare Safety Network (NHSN) surveillance definitions, we considered all bloodstream isolates as representative of infection and used modified definitions as in reference 7 to classify urinary and respiratory cultures as representative of infection versus colonization (Table S2) (7, 39). Any isolate that did not meet the criteria for infection was classified as colonization. We did not incorporate physician interpretation in applying the criteria to ensure consistency in applying the definition.

10.1128/mSystems.00177-21.8 TABLE S2 Definitions of infection for urinary and respiratory cultures. Download Table S2, DOCX file, 0.01 MB. Copyright © 2021 Lapp et al. 2021 Lapp et al. https://creativecommons.org/licenses/by/4.0/ This content is distributed under the terms of the Creative Commons Attribution 4.0 International license.

10.1128/mSystems.00177-21.9 TABLE S3 Bivariable analysis of patient features associated with CRKP ST258 infection in LTACH residents. Download Table S3, DOCX file, 0.02 MB. Copyright © 2021 Lapp et al. 2021 Lapp et al. https://creativecommons.org/licenses/by/4.0/ This content is distributed under the terms of the Creative Commons Attribution 4.0 International license.

### Feature sets.

We studied the association between five different feature sets and infection/colonization in CRKP ST258 (Fig. S1C); the feature sets are described below. See the supplemental material for details on feature set creation and processing. Counts below are for confident features from the entire data set prior to subsetting for different analyses. Feature sets are as follows: (i) patient—clinical features described in the work of Han et al. (*n* = 50; Table S3) (15); (ii) uncurated genomic—single nucleotide variants, indels, insertion sequence elements, and accessory genes (*n* = 2,447); (iii) uncurated grouped genomic—variants grouped into genes (i.e., a burden test, e.g., reference 17) and accessory genes (*n* = 3,159); (iv) curated genomic—features identified by Kleborate (18), a tool designed to identify the presence of various genes and mutations known to be associated with either CRKP virulence or antibiotic resistance (*n* = 91); (v) patient and curated genomic—patient features and curated genomic features (*n* = 141).

### Machine learning and model selection.

We aimed to classify clinical infection (versus colonization) using each of the different feature sets (see above); we built classifiers using the first clinical isolate from each patient for all isolates, only respiratory isolates, and only urinary isolates. We performed L2 regularized logistic regression on all feature sets using a modified version of the machine learning pipeline presented in the work of Topçuoğlu et al. (40) using caret version 6.0-85 (41) in R version 3.6.2 (42) (Fig. S1D1). Furthermore, for the patient and curated genomic feature set we performed elastic net, random forest, and support vector machine with a radial basis kernel using the same method but implemented in mikropml version 0.0.2 (43). We randomly split the data into 100 unique ∼80/20 train/test splits, keeping all isolates from each LTACH grouped in either the training set or the held-out test set to control for facility-level differences among the isolates (e.g., background of circulating strains within each facility, patient population, and clinician test ordering frequency). For valid comparison, the train/test splits were identical across models generated with different feature sets. Hyperparameters were selected via cross-validation on the training set to maximize the average AUROC across cross-validation folds. See the supplemental material for more details.

### Model performance.

We measured model performance using the median test area under the receiver operating characteristic curve (AUROC) and area under the precision recall curve (AUPRC), as well as the interquartile range, across all 100 train/test splits (Fig. S1D2).

### Features consistently associated with colonization or infection.

To determine the importance of each feature in predicting colonization versus infection, we measured how much each feature influenced model performance by calculating feature importance using a permutation test (40) (Fig. S1D3). For each combination of feature and data split, we randomly permuted the feature and calculated the “permuted test AUROC” using the model generated with the training data. Features with a correlation of 1 were permuted together. We performed this permutation test 100 times for each feature/data split pair and obtained a mean feature importance for each data split. A mean feature importance above zero indicates that that feature improved model performance for that data split. We highlight features where the mean permuted test AUROC was above zero in at least 75% of the data splits. In this way, the permutation importance method allows us to take into account the variation we observe across the 100 models, which is not possible with standard parametric statistical tests or odds ratios.

### Insertion sequence identification.

We identified insertion sequences in the *kfoC* gene by running panISa on reads aligned to a reference genome (34, 35, 44–46). See the supplemental material for more details.

### Data analysis and visualization.

See the supplemental material for details on data analysis and visualization in R version 3.6.2 (42, 47–51). All code and data that are not protected health information are on GitHub (https://github.com/Snitkin-Lab-Umich/ml-crkp-infection-manuscript).

10.1128/mSystems.00177-21.1 TEXT S1 Additional details on clinical data extraction, genomic data processing, and analytical pipelines. Download Text S1, DOCX file, 0.02 MB. Copyright © 2021 Lapp et al. 2021 Lapp et al. https://creativecommons.org/licenses/by/4.0/ This content is distributed under the terms of the Creative Commons Attribution 4.0 International license.
