# Supplementary material for: Patient and Microbial Genomic Factors Associated with Carbapenem-Resistant Klebsiella pneumoniae Extraintestinal Colonization and Infection
Source: mSystems. 2021 Mar 16;6(2):e00177-21. doi: 10.1128/mSystems.00177-21 (PMC8546970; doi:10.1128/mSystems.00177-21)
Supplement: TABLE S1 [file msystems.00177-21-st001.docx]

| **Sequence Type** | **Colonization** | | **Infection** | | | **Total (%)** |
| --- | --- | --- | --- | --- | --- | --- |
|  | **Respiratory** | **Urinary** | **Blood** | **Respiratory** | **Urinary** |  |
| ST15 | 4 | 3 | 1 | 2 | 0 | 10 (2.8) |
| ST15-1LV | 0 | 0 | 0 | 1 | 0 | 1 (0.3) |
| ST17 | 0 | 0 | 0 | 2 | 0 | 2 (0.6) |
| ST2237 | 0 | 1 | 0 | 0 | 0 | 1 (0.3) |
| ST258 | 121 | 71 | 28 | 62 | 49 | 331 (93.2) |
| ST307 | 1 | 4 | 0 | 2 | 2 | 9 (2.5) |
| ST36 | 1 | 0 | 0 | 0 | 0 | 1 (0.3) |
