# Supplementary figures and images for: Patient and Microbial Genomic Factors Associated with Carbapenem-Resistant Klebsiella pneumoniae Extraintestinal Colonization and Infection
Source: mSystems. 2021 Mar 16;6(2):e00177-21. doi: 10.1128/mSystems.00177-21 (PMC8546970; doi:10.1128/mSystems.00177-21)

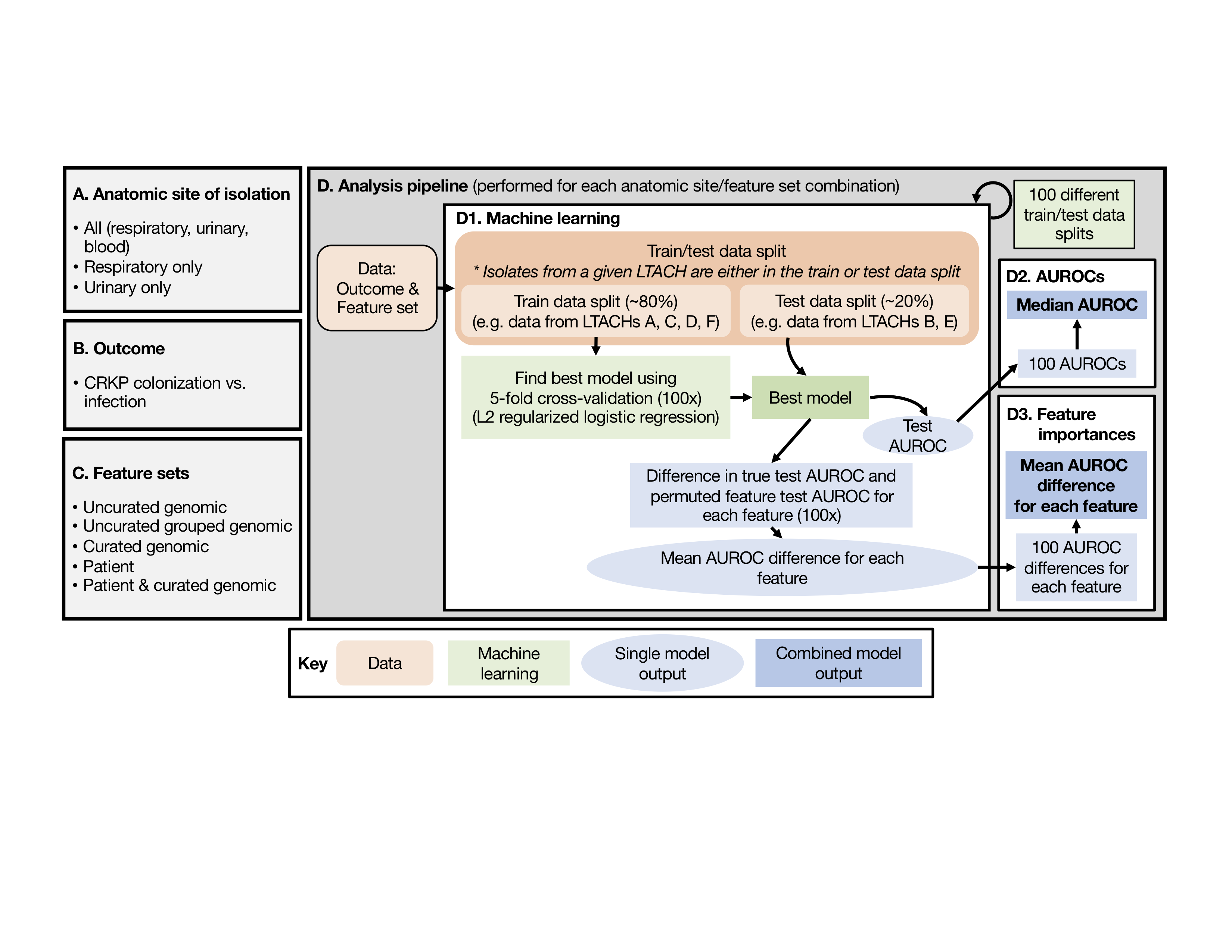

Supplement: FIG S1 [file msystems.00177-21-sf001.tif]

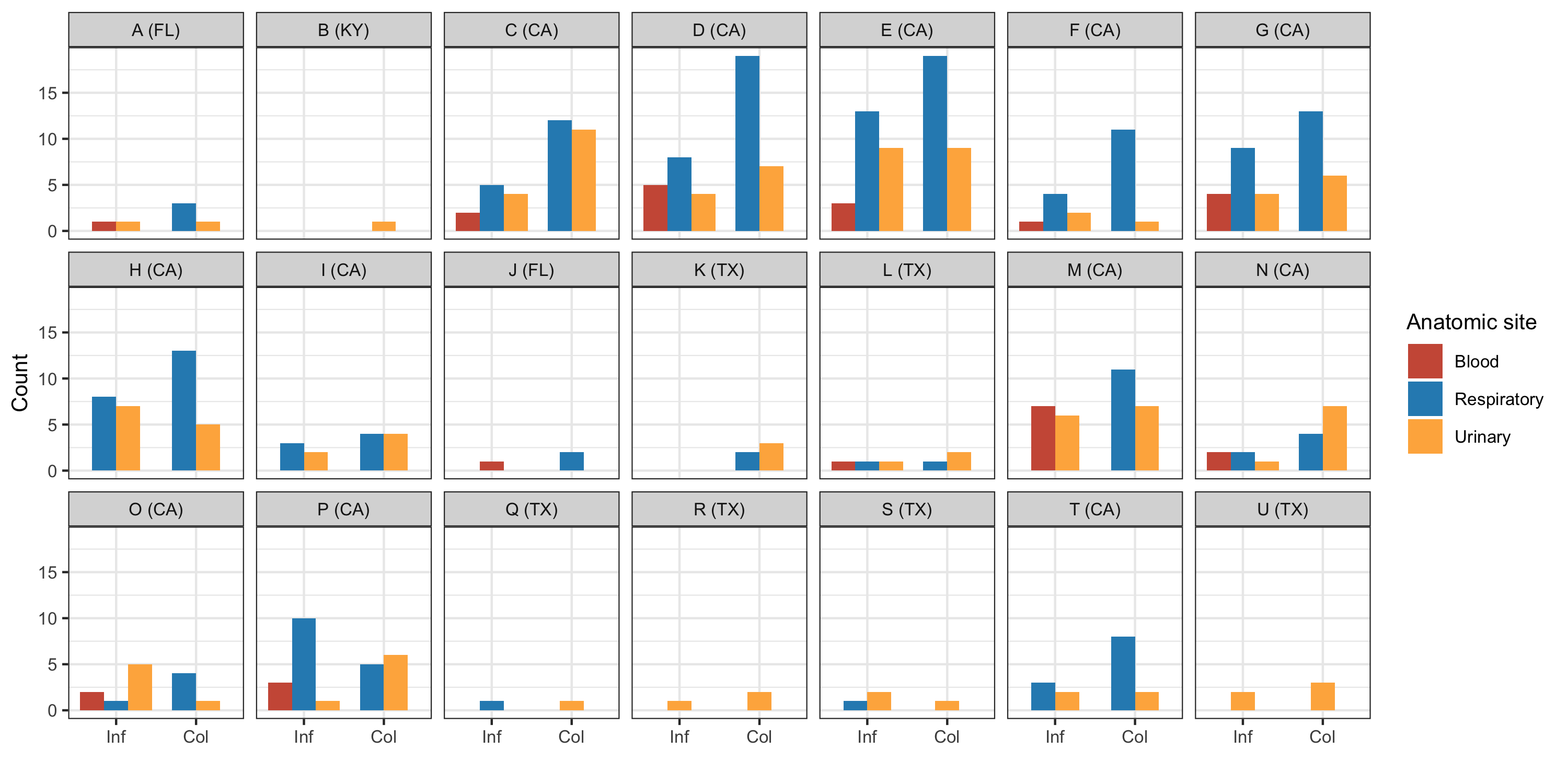

Supplement: FIG S2 [file msystems.00177-21-sf002.tif]

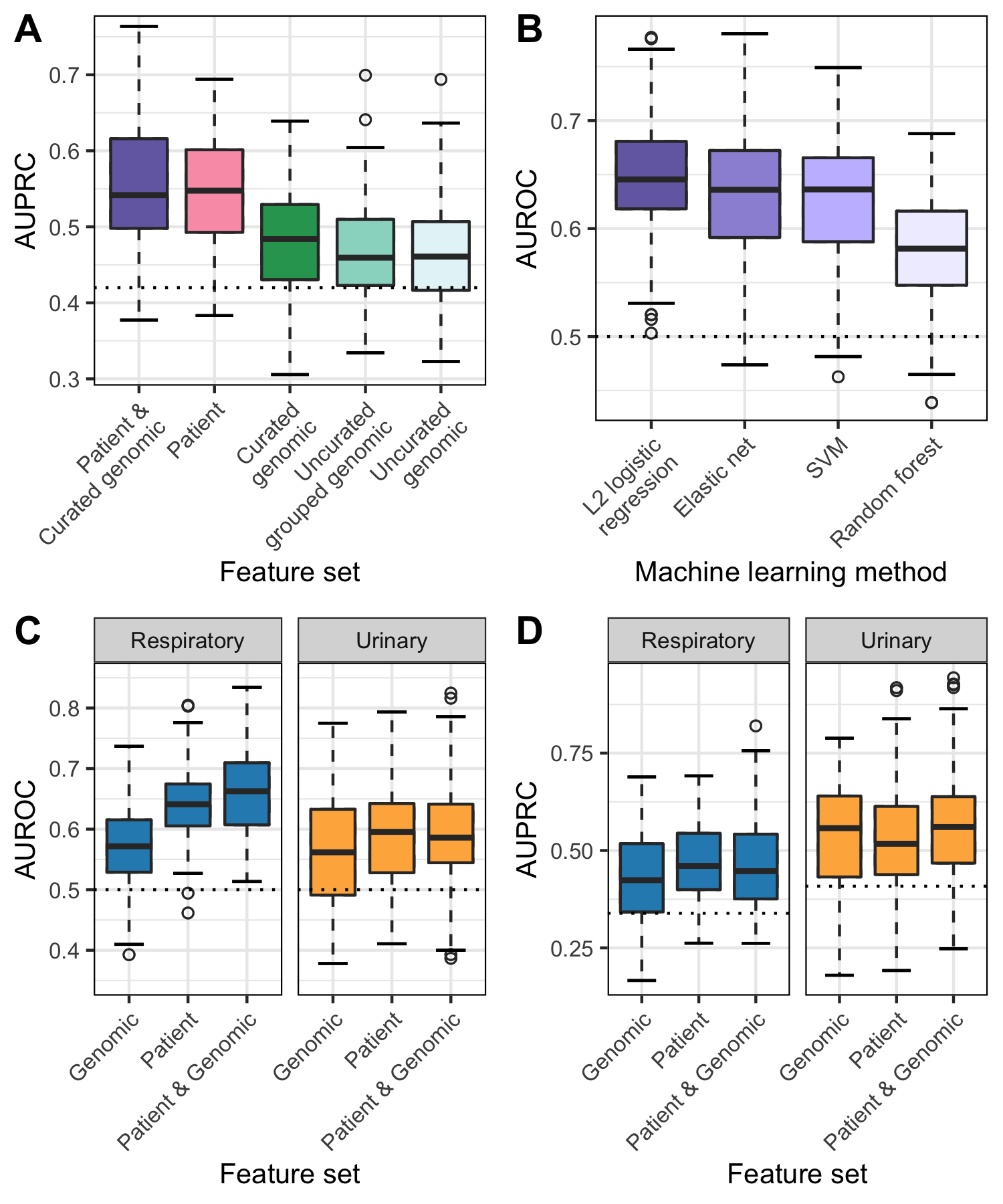

Supplement: FIG S3 [file msystems.00177-21-sf003.tif]

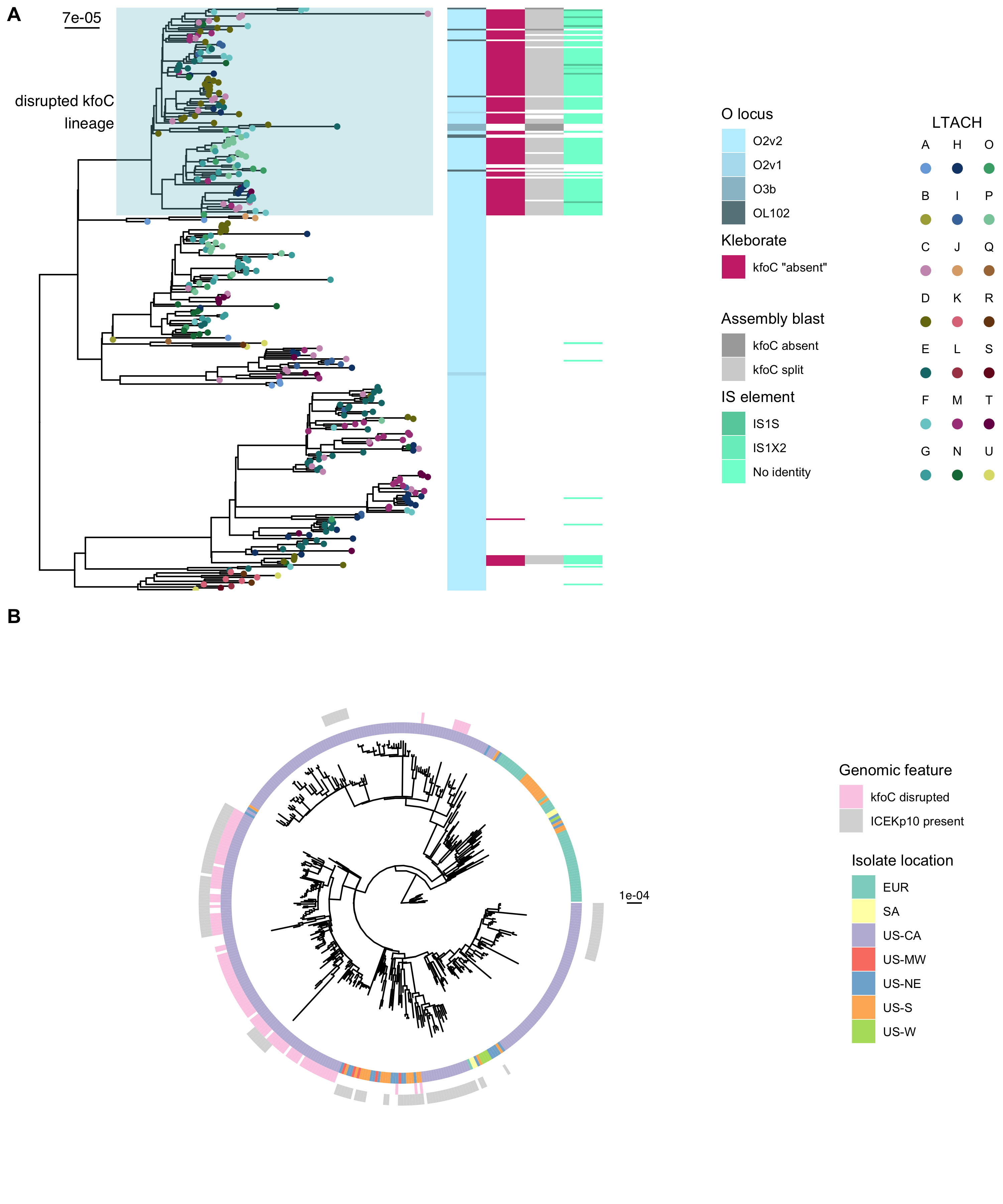

Supplement: FIG S5 [file msystems.00177-21-sf005.tif]
