# Supplementary material for: Patient and Microbial Genomic Factors Associated with Carbapenem-Resistant Klebsiella pneumoniae Extraintestinal Colonization and Infection
Source: mSystems. 2021 Mar 16;6(2):e00177-21. doi: 10.1128/mSystems.00177-21 (PMC8546970; doi:10.1128/mSystems.00177-21)
Supplement: TABLE S2 [file msystems.00177-21-st002.docx]

| **Source** | **Infection definition** |
| --- | --- |
| Urinary | ≥100,000 colony-forming units/mL on urinary culture with no more than 2 species of microorganisms AND at least one of the following:   - Leukocytosis (≥10,000 cells/µL) ≤ 7 days prior to specimen collection - Positive urinalysis demonstrated by at least one of the following findings:   - Positive dipstick for leukocyte esterase and/or nitrate   - Pyuria (urinary specimen with ≥ 10 white blood cells/mL or ≥ 3 white blood cells/high power field of unspun urinary)   - Microorganisms seen on Gram stain of unspun urinary |
| Respiratory | Chest X-ray with interpretation of pneumonia, probable pneumonia, or infiltrate/consolidation AND leukocytosis (≥10,000 cells/µL) ≤ 7 days prior to specimen collection |
