# Supplementary material for: Patient and Microbial Genomic Factors Associated with Carbapenem-Resistant Klebsiella pneumoniae Extraintestinal Colonization and Infection
Source: mSystems. 2021 Mar 16;6(2):e00177-21. doi: 10.1128/mSystems.00177-21 (PMC8546970; doi:10.1128/mSystems.00177-21)
Supplement: TABLE S3 [file msystems.00177-21-st003.docx]

| **Variable** | **Infection (n=139)** | **Colonization (n=192)** | **All** | | **Respiratory** | | **Urinary** | |
| --- | --- | --- | --- | --- | --- | --- | --- | --- |
|  |  |  | **OR (95% CI)** | **P value** | **OR (95% CI)** | **P value** | **OR (95% CI)** | **P value** |
| Blood culture | 28 (20%) | 0 (0%) | - | - | - | - | - | - |
| Respiratory culture | 62 (45%) | 121 (63%) | 0.47 (0.3-0.76) | 0.0011 | - | - | - | - |
| Urinary culture | 49 (35%) | 71 (37%) | 0.93 (0.57-1.5) | 0.82 | - | - | - | - |
| Admission to an LTACH in the year prior to culture^1^ | 9 (6%) | 7 (4%) | 1.83 (0.59-5.93) | 0.30 | 2.03 (0.45-9.19) | 0.31 | 2.23 (0.25-27.73) | 0.40 |
| Length of stay before culture, median (IQR)^2^ | 23 (9.0-50) | 18 (2.0-34) | - | 0.98 | - | 0.66 | - | 0.88 |
| Age, median (IQR)^2^ | 72 (64-79) | 73 (64-83) | - | 0.029 | - | 0.0010 | - | 0.55 |
| Sex (male) | 74 (53%) | 102 (53%) | 1 (0.63-1.59) | 1.0 | 1.15 (0.59-2.29) | 0.75 | 0.94 (0.42-2.1) | 1.0 |
| Presence of a central venous catheter | 87 (63%) | 97 (51%) | 1.64 (1.03-2.62) | 0.033 | 3.36 (1.54-7.87) | 0.00094 | 0.79 (0.34-1.81) | 0.57 |
| Tracheostomy | 83 (60%) | 141 (73%) | 0.54 (0.33-0.88) | 0.0091 | 1.45 (0.41-6.51) | 0.78 | 0.42 (0.17-0.99) | 0.035 |
| Presence of a urinary catheter | 87 (63%) | 94 (49%) | 1.74 (1.09-2.79) | 0.019 | 4.19 (2.06-8.88) | 0.000020 | 0.71 (0.32-1.59) | 0.45 |
| Congestive heart failure | 27 (19%) | 38 (20%) | 0.98 (0.54-1.75) | 1.0 | 1.74 (0.8-3.76) | 0.14 | 0.25 (0.04-0.95) | 0.035 |
| Acute or chronic respiratory failure | 55 (40%) | 66 (34%) | 1.25 (0.77-2.01) | 0.36 | 1.93 (0.99-3.78) | 0.040 | 0.68 (0.27-1.64) | 0.42 |
| Acute kidney injury | 76 (55%) | 76 (40%) | 1.84 (1.16-2.93) | 0.0074 | 2.65 (1.36-5.29) | 0.0028 | 1.18 (0.53-2.62) | 0.71 |
| Malignancy (solid or liquid) | 16 (12%) | 27 (14%) | 0.8 (0.38-1.61) | 0.51 | 0.97 (0.31-2.76) | 1.0 | 0.62 (0.18-1.94) | 0.45 |
| Brain injury^3^ | 31 (22%) | 31 (16%) | 1.49 (0.82-2.7) | 0.20 | 3.37 (1.57-7.32) | 0.00082 | 0.1 (0-0.75) | 0.014 |
| Presence of a gastrostomy tube | 62 (45%) | 59 (31%) | 1.81 (1.12-2.93) | 0.011 | 2.98 (1.52-5.94) | 0.00083 | 1.45 (0.61-3.44) | 0.42 |
| Obesity^4^ | 4 (3%) | 14 (7%) | 0.38 (0.09-1.24) | 0.091 | 0.23 (0.01-1.81) | 0.28 | 0.46 (0.04-2.74) | 0.47 |
| Malnourished/underweight^5^ | 38 (27%) | 43 (22%) | 1.3 (0.76-2.22) | 0.30 | 2.02 (0.94-4.32) | 0.065 | 0.66 (0.24-1.67) | 0.39 |
| Transplant^6^ | 4 (3%) | 3 (2%) | 1.86 (0.31-12.93) | 0.46 | 3.01 (0.33-36.91) | 0.34 | 1.45 (0.02-115.98) | 1.0 |
| Cirrhosis | 8 (6%) | 6 (3%) | 1.89 (0.56-6.77) | 0.28 | 3.43 (0.64-22.82) | 0.12 | 0.47 (0.01-6.12) | 0.64 |
| Severe chronic kidney disease^7^ | 52 (37%) | 55 (29%) | 1.49 (0.91-2.43) | 0.097 | 2.12 (1.08-4.19) | 0.023 | 1.06 (0.42-2.63) | 1.0 |
| Pulmonary disease^8^ | 25 (18%) | 34 (18%) | 1.02 (0.55-1.87) | 1.0 | 1.09 (0.44-2.6) | 0.84 | 0.68 (0.21-1.99) | 0.48 |
| Ventilator-dependent respiratory failure | 52 (37%) | 48 (25%) | 1.79 (1.09-2.96) | 0.021 | 3.34 (1.69-6.69) | 0.00023 | 0.49 (0.11-1.78) | 0.27 |
| Stage IV/V decubitus ulcer | 31 (22%) | 32 (17%) | 1.43 (0.79-2.58) | 0.20 | 2.02 (0.94-4.32) | 0.065 | 1.54 (0.5-4.81) | 0.44 |
| Amikacin^9^ | 23 (17%) | 14 (7%) | 2.51 (1.18-5.52) | 0.013 | 1.88 (0.63-5.49) | 0.21 | 2.77 (0.66-13.69) | 0.12 |
| Aztreonam^9^ | 5 (4%) | 6 (3%) | 1.16 (0.27-4.65) | 1.0 | 1.99 (0.26-15.34) | 0.41 | 0 (0-3.49) | 0.27 |
| Trimethoprim/sulfamethoxazole^9^ | 6 (4%) | 5 (3%) | 1.68 (0.42-7.13) | 0.54 | 1.99 (0.26-15.34) | 0.41 | 1.46 (0.1-20.84) | 1.0 |
| Cefepime^9^ | 20 (14%) | 32 (17%) | 0.84 (0.43-1.6) | 0.65 | 1.1 (0.42-2.73) | 0.83 | 0.57 (0.17-1.74) | 0.33 |
| Ceftaroline^9^ | 4 (3%) | 3 (2%) | 1.86 (0.31-12.93) | 0.46 | 0 (0-4.73) | 0.55 | Inf (0.61-Inf) | 0.066 |
| Ceftazidime^9^ | 3 (2%) | 7 (4%) | 0.58 (0.1-2.61) | 0.53 | 0.97 (0.15-4.76) | 1.0 | 0 (0-56.46) | 1.0 |
| Ceftriaxone^9^ | 11 (8%) | 8 (4%) | 1.97 (0.7-5.82) | 0.16 | 1.68 (0.39-6.91) | 0.51 | 3.04 (0.42-34.89) | 0.22 |
| Ciprofloxacin^9^ | 2 (1%) | 6 (3%) | 0.45 (0.04-2.59) | 0.48 | 1.96 (0.02-155.49) | 1.0 | 0 (0-1.55) | 0.078 |
| Colistin^9^ | 12 (9%) | 11 (6%) | 1.55 (0.61-4.02) | 0.38 | 2.4 (0.72-8.22) | 0.15 | 0.35 (0.01-3.7) | 0.65 |
| Daptomycin^9^ | 10 (7%) | 3 (2%) | 4.86 (1.22-28) | 0.018 | 8.18 (0.79-409.93) | 0.046 | 4.75 (0.8-50.2) | 0.062 |
| Ertapenem^9^ | 6 (4%) | 4 (2%) | 2.12 (0.49-10.4) | 0.33 | 3.97 (0.2-237.55) | 0.27 | 0.47 (0.01-6.12) | 0.64 |
| Metronidazole^9^ | 25 (18%) | 35 (18%) | 0.98 (0.53-1.8) | 1.0 | 0.97 (0.43-2.11) | 1.0 | 1.27 (0.33-4.77) | 0.77 |
| Gentamicin^9^ | 5 (4%) | 5 (3%) | 1.39 (0.31-6.19) | 0.75 | 0.98 (0.09-7.03) | 1.0 | 2.95 (0.15-178) | 0.57 |
| Imipenem^9^ | 3 (2%) | 2 (1%) | 2.09 (0.24-25.36) | 0.65 | 1.96 (0.02-155.49) | 1.0 | 0 (0-56.46) | 1.0 |
| Levofloxacin^9^ | 18 (13%) | 25 (13%) | 0.99 (0.49-1.99) | 1.0 | 1.57 (0.63-3.85) | 0.29 | 0.61 (0.13-2.37) | 0.56 |
| Linezolid^9^ | 23 (17%) | 22 (11%) | 1.53 (0.77-3.03) | 0.20 | 1.47 (0.54-3.83) | 0.49 | 1.1 (0.29-3.91) | 1.0 |
| Meropenem^9^ | 39 (28%) | 50 (26%) | 1.11 (0.66-1.86) | 0.71 | 1.13 (0.54-2.31) | 0.73 | 0.88 (0.32-2.33) | 0.83 |
| Polymyxin^9^ | 8 (6%) | 4 (2%) | 2.86 (0.75-13.26) | 0.13 | 0.98 (0.09-7.03) | 1.0 | Inf (0.27-Inf) | 0.16 |
| Tigecycline^9^ | 25 (18%) | 17 (9%) | 2.25 (1.11-4.66) | 0.019 | 2.63 (1.04-6.75) | 0.026 | 2.55 (0.68-10.63) | 0.14 |
| Tobramycin^9^ | 7 (5%) | 10 (5%) | 0.97 (0.3-2.89) | 1.0 | 0.47 (0.05-2.47) | 0.50 | 2.23 (0.25-27.73) | 0.40 |
| Piperacillin/tazobactam^9^ | 18 (13%) | 23 (12%) | 1.09 (0.53-2.22) | 0.87 | 0.9 (0.29-2.52) | 1.0 | 1.53 (0.46-5.09) | 0.43 |
| Intravenous vancomycin^9^ | 52 (37%) | 62 (32%) | 1.25 (0.77-2.03) | 0.35 | 1.07 (0.54-2.09) | 0.87 | 1.03 (0.4-2.6) | 1.0 |
| Aminoglycoside^9^ | 32 (23%) | 27 (14%) | 1.82 (1-3.36) | 0.042 | 1.14 (0.47-2.66) | 0.84 | 2.75 (0.83-9.98) | 0.099 |
| Polymyxin or colistin^9^ | 20 (14%) | 15 (8%) | 1.98 (0.92-4.34) | 0.070 | 1.92 (0.68-5.33) | 0.22 | 1.09 (0.15-6.78) | 1.0 |
| Third-generation cephalosporin^9^ | 14 (10%) | 15 (8%) | 1.32 (0.57-3.05) | 0.56 | 1.34 (0.45-3.82) | 0.62 | 2 (0.32-14.33) | 0.44 |
| Anti-pseudomonal antibiotic^9,10^ | 88 (63%) | 115 (60%) | 1.15 (0.72-1.86) | 0.57 | 1.24 (0.63-2.49) | 0.52 | 0.76 (0.34-1.69) | 0.58 |
| Fluoroquinolone^9^ | 20 (14%) | 31 (16%) | 0.87 (0.45-1.67) | 0.76 | 1.62 (0.67-3.86) | 0.29 | 0.36 (0.08-1.27) | 0.12 |
| Carbapenem^9^ | 59 (42%) | 68 (35%) | 1.34 (0.84-2.16) | 0.21 | 1.26 (0.64-2.45) | 0.52 | 0.98 (0.41-2.31) | 1.0 |
| Piperacillin/tazobactam or ceftazidime^9^ | 20 (14%) | 29 (15%) | 0.94 (0.48-1.82) | 0.88 | 0.86 (0.32-2.14) | 0.83 | 1.34 (0.41-4.28) | 0.60 |
| ^1^LTACH=long-term acute care hospital  ^2^IQR=interquartile range  ^3^Anoxic brain injury or cerebrovascular accident (CVA) causing paresis  ^4^BMI ≥30 kg/m^2  ^5^BMI ≤18.5 kg/m^2  ^6^Solid organ transplant or hematopoeitic stem cell transplant  ^7^Stage IV or dialysis  ^8^Chronic obstructive pulmonary disease (COPD) or chronic bronchitis  ^9^Receipt for ≥48 hours in the 30 days prior to culture  ^10^Aminoglycosides, cefepime, ceftazidime, fluoroquinolones, polymyxins, carbapenems, or piperacillin-tazobactam | | | | | | | | |
|  |  |  |  |  |  |  |  |  |
|  |  |  |  |  |  |  |  |  |
|  |  |  |  |  |  |  |  |  |
|  |  |  |  |  |  |  |  |  |
