# Supplementary material for: Patient and Microbial Genomic Factors Associated with Carbapenem-Resistant Klebsiella pneumoniae Extraintestinal Colonization and Infection
Source: mSystems. 2021 Mar 16;6(2):e00177-21. doi: 10.1128/mSystems.00177-21 (PMC8546970; doi:10.1128/mSystems.00177-21)
Supplement: TEXT S1 [file msystems.00177-21-s0001.docx]

**Supplementary materials**

**Supplementary methods**

**Clinical data**

The majority of data elements were obtained from the electronic health record (EHR) which captures all data in the medical record across clinical sites. This EHR-based electronic database contains all demographic data, laboratory data (including microbiology), medication data, and radiographic data. ICD diagnostic and procedure codes were used to ascertain all coded primary and secondary diagnoses and procedures. The same EHR is used at all sites and is fully integrated into the EHR-based relational database. We chose not to include inflammatory markers (e.g. white blood cell count, procalcitonin, etc.) as they are highly non-specific and add little to designate a culture as representing infection vs. colonization, particularly in patients who typically have numerous comorbidities contributing to their acute illness.

**Pipeline**

We created a snakemake pipeline (1) to perform data pre-processing, machine learning analysis, and figure generation.

**Genomic data**

We used Kleborate version 0.3.0 (2) to identify sequence type, capsular types (K locus (3) and O locus (4)), virulence factor (5), and antibiotic resistance genes. In addition, we identified single nucleotide variants (SNVs) and indels by mapping reads to the KPNIH1 reference genome (BioProject accession number PRJNA73191; https://github.com/Snitkin-Lab-Umich/variant_calling_pipeline (6)). After variant calling, we identified the predicted functional impact of variants using SnpEff version 4.3T (7). We also identified large insertion sequence (IS) elements using panISa version 0.1.4 with the default settings (8), and accessory genome genes using Roary version 3.12.0 with the default settings (9).

**Phylogenetic tree reconstruction**

We constructed a phylogenetic tree of all ST258 isolates by first creating a whole-genome alignment by mapping reads to the KPNIH1 reference genome (GenBank accession number CP008827.1) using bwa mem version 0.7.12 (10); samtools version 1.9 (11) was used to generate binary alignment files. We then masked sites identified as recombinant by Gubbins version 2.3.2 (12) and used this masked whole-genome alignment to build a maximum likelihood phylogeny with IQ-TREE version 1.6.12 (13) using a GTR model of nucleotide substitution and ultra-fast bootstrap with 1000 replicates (-b 1000) (14). The tree was midpoint-rooted using the midpoint.root function in phytools version 0.6-99 (15), thus splitting the tree into ST258 clades I and II (16).

**Phylogenetic clustering test**

To determine whether infection isolates cluster on the phylogeny more than expected by random chance, we performed a permutation test (17). To identify the true number of isolates in a pure cluster while controlling for isolate clustering by LTACH, we enumerated isolates in pure infection or colonization subtrees that also contain isolates from more than one LTACH. Then we randomized the infection labels across isolates 1000 times and enumerated the number of isolates in a pure subtree for each randomization. We then compared the random counts of isolates in a pure cluster to the true number and calculated an empirical p-value.

**Genomic feature set creation**

To determine how well machine learning models perform using a targeted vs. untargeted approach, we performed machine learning with uncurated genomic features as well as curated genomic features known to be related to virulence. In an attempt to increase power, we not only performed machine learning on individual uncurated genomic features, but also using a burden test where genomic variants were grouped into genes and features were presence/absence of a variant in a gene. This may help capture variation associated with infection at the gene level that might not be captured at the variant level (e.g. different samples can have different variants in the same gene that all lead to a similar function). For the uncurated grouped genomic analysis, grouped variants included SNVs and indels identified as moderate or high impact by SnpEff and IS elements in a gene or upstream of a gene. We also grouped modifier variants identified by SnpEff into intergenic regions. In addition to core genes, we included accessory genes identified by Roary (9) as binary categorical features in both of the uncurated feature sets.

**Feature set pre-processing**

We used five feature sets to study the association between infection and colonization. We preprocessed all five datasets by mapping categorical features to binary variables, centering and scaling the continuous features (age and length of stay) to a mean of zero and a variance of one, and removing features present in only one sample or all but one sample. Some antibiotic features were antibiotic classes, and thus aggregates of other features (e.g. aminoglycoside, see Table S3). For the uncurated genomic and uncurated grouped genomic datasets we collapsed genomic features with an identical pattern across all isolates to reduce machine learning runtime.

**Machine learning rationale**

We chose to perform L2 regularized logistic regression because it is easily interpretable, often performs just as well as more complicated methods when limited training data are available (18), and has a grouping effect, which means that all associated features are identified even if they are collinear (19). L2 regularization is equivalent to including a zero-mean Gaussian prior on the weights. Thus, L2 regularized logistic regression includes implicit feature selection of sparse data by reducing the majority of feature weights to zero. Furthermore, this method allows us to maintain correlated features in the model, which aids in our interpretation of results as there may be features that together are important for understanding differences in infection and virulence. Finally, we found that L2 regularized logistic regression performs as well as other machine learning models for our dataset (**Figure S3**), and that hyperparameter tuning for elastic net leads to a more L2-like model rather than an L1-like model.

**Machine learning details**

The machine learning pipeline sets at least 20% of the samples aside for testing in each train/test split. We split the training data into train and validate sets by LTACH using the groupKMultiFolds function from the caret R package (caret version 6.0-85) (20). Within each training set, we selected hyperparameters via five-fold cross-validation 100 times, maximizing the average cross-validation AUROC.

**Comparing model performance between feature sets and methods**

To determine whether there was a significant difference in model performance between different feature sets and different machine learning methods, we calculated a two-sided empirical p-value for each identical train/test split using the formula 2 x min(fraction of AUROC differences ≥ 0, fraction of AUROC differences ≤ 0) (18).

**Identification of IS element insertions in certain O2v2 kfoC genes**

One of the curated genomic features we identified as associated with infection was what Kleborate called a missing gene (kfoC) in the O locus operon of certain O2v2 serotypes. On the Kleborate website, they indicate that a missing gene could represent a truly missing gene, or a gene that is split between two different contigs. To further investigate this, we used blastn version 2.9.0 (21) to search for kfoC genes in each of the samples. Additionally, we aligned the reads of all genomes to CP031810, a complete *K. pneumoniae* genome from PATRIC (22) using bwa mem version 0.7.12 (10), and samtools (11) was used to generate binary alignment files. Next, we used panISa (8) to identify IS element insertions in kfoC.

**Data analysis & visualization**

We used Fisher’s exact tests for bivariable analyses with categorical variables, and Wilcoxon rank-sum tests for bivariable analyses with continuous variables. We performed all data analysis and visualization in R version 3.6.2 (23) using the following packages: tidyverse version 1.3.0 (24), cowplot version 1.0.0 (25), ggtree version 2.0.1 (26, 27), ape version 5.3 (28), phytools version 0.6-99 (15), and grid version 3.6.2 (29).

**Supplementary results**

**Patients with different sequence types show no substantive differences in infection status, anatomic site of isolation, or clinical characteristics**

Over 90% of the isolates in our dataset were ST258 (**Table S2**), the dominant strain in the US (30). At the level of sequence type, we found no difference in infection prevalence (p=0.44) or anatomic site of isolation (p=0.66), indicating that at this coarse level there was not evidence of differences in strain virulence or adaptation to a certain anatomic site. Bivariable comparison of patient factors between patients with different sequence types revealed only four significant differences (unadjusted p-values < 0.05; previous use of piperacillin/tazobactam, cefepime, ciprofloxacin, or fluoroquinolones). As we found no substantive differences in infection, anatomic site of isolation, or patient variables when comparing different sequence types, we chose to focus all subsequent analyses on ST258, the dominant sequence type in our dataset. The genetic variation of isolates within ST258 is much smaller than the genetic variation between sequence types, so limiting our analyses to ST258 could improve our ability to identify associations of interest within ST258.

**Supplementary references**

1.     Köster J, Rahmann S. 2012. Snakemake—a scalable bioinformatics workflow engine. Bioinformatics 28:2520–2522.

2.     Holt K. 2020. katholt/Kleborate. Python.

3.     Wyres KL, Wick RR, Gorrie C, Jenney A, Follador R, Thomson NR, Holt KE. 2016. Identification of Klebsiella capsule synthesis loci from whole genome data. Microbial Genomics, 2:e000102.

4.     Wick RR, Heinz E, Holt KE, Wyres KL. 2018. Kaptive Web: User-Friendly Capsule and Lipopolysaccharide Serotype Prediction for Klebsiella Genomes. Journal of Clinical Microbiology 56.

5.     Lam MMC, Wick RR, Wyres KL, Gorrie CL, Judd LM, Jenney AWJ, Brisse S, Holt KE. 2018. Genetic diversity, mobilisation and spread of the yersiniabactin-encoding mobile element ICEKp in Klebsiella pneumoniae populations. Microbial Genomics, 4:e000196.

6.     Conlan S, Park M, Deming C, Thomas PJ, Young AC, Coleman H, Sison C, Program NCS, Weingarten RA, Lau AF, Dekker JP, Palmore TN, Frank KM, Segre JA. 2016. Plasmid Dynamics in KPC-Positive Klebsiella pneumoniae during Long-Term Patient Colonization. mBio

7.     Cingolani P, Platts A, Wang LL, Coon M, Nguyen T, Wang L, Land SJ, Lu X, Ruden DM. 2012. A program for annotating and predicting the effects of single nucleotide polymorphisms, SnpEff. Fly (Austin) 6:80–92.

8.     Treepong P, Guyeux C, Meunier A, Couchoud C, Hocquet D, Valot B. 2018. panISa: ab initio detection of insertion sequences in bacterial genomes from short read sequence data. Bioinformatics 34:3795–3800.

9.     Page AJ, Cummins CA, Hunt M, Wong VK, Reuter S, Holden MTG, Fookes M, Falush D, Keane JA, Parkhill J. 2015. Roary: rapid large-scale prokaryote pan genome analysis. Bioinformatics 31:3691–3693.

10.     Li H, Durbin R. 2009. Fast and accurate short read alignment with Burrows–Wheeler transform. Bioinformatics 25:1754–1760.

11.     Li H, Handsaker B, Wysoker A, Fennell T, Ruan J, Homer N, Marth G, Abecasis G, Durbin R. 2009. The Sequence Alignment/Map format and SAMtools. Bioinformatics 25:2078–2079.

12.     Croucher NJ, Page AJ, Connor TR, Delaney AJ, Keane JA, Bentley SD, Parkhill J, Harris SR. 2015. Rapid phylogenetic analysis of large samples of recombinant bacterial whole genome sequences using Gubbins. Nucleic Acids Res 43:e15–e15.

13.     Nguyen L-T, Schmidt HA, von Haeseler A, Minh BQ. 2015. IQ-TREE: A Fast and Effective Stochastic Algorithm for Estimating Maximum-Likelihood Phylogenies. Mol Biol Evol 32:268–274.

14.     Minh BQ, Nguyen MAT, von Haeseler A. 2013. Ultrafast Approximation for Phylogenetic Bootstrap. Mol Biol Evol 30:1188–1195.

15.     Revell LJ. 2012. phytools: an R package for phylogenetic comparative biology (and other things). Methods in Ecology and Evolution 3:217–223.

16.     DeLeo FR, Chen L, Porcella SF, Martens CA, Kobayashi SD, Porter AR, Chavda KD, Jacobs MR, Mathema B, Olsen RJ, Bonomo RA, Musser JM, Kreiswirth BN. 2014. Molecular dissection of the evolution of carbapenem-resistant multilocus sequence type 258 Klebsiella pneumoniae. PNAS 111:4988–4993.

17.     Popovich KJ, Snitkin ES, Hota B, Green SJ, Pirani A, Aroutcheva A, Weinstein RA. 2017. Genomic and Epidemiological Evidence for Community Origins of Hospital-Onset Methicillin-Resistant Staphylococcus aureus Bloodstream Infections. J Infect Dis 215:1640–1647.

18.     Topçuoğlu BD, Lesniak NA, Ruffin M, Wiens J, Schloss PD. 2019. Effective application of machine learning to microbiome-based classification problems. bioRxiv 816090.

19.     Schreiber-Gregory DN, Waller J, Smith T. 2018. Ridge Regression and multicollinearity: An in-depth review. Model Assisted Statistics & Applications 13:359–365.

20.     Kuhn M. 2008. Building Predictive Models in R Using the caret Package. 1. Journal of Statistical Software 28:1–26.

21.     Camacho C, Coulouris G, Avagyan V, Ma N, Papadopoulos J, Bealer K, Madden TL. 2009. BLAST+: architecture and applications. BMC Bioinformatics 10:421.

22.     Wattam AR, Davis JJ, Assaf R, Boisvert S, Brettin T, Bun C, Conrad N, Dietrich EM, Disz T, Gabbard JL, Gerdes S, Henry CS, Kenyon RW, Machi D, Mao C, Nordberg EK, Olsen GJ, Murphy-Olson DE, Olson R, Overbeek R, Parrello B, Pusch GD, Shukla M, Vonstein V, Warren A, Xia F, Yoo H, Stevens RL. 2017. Improvements to PATRIC, the all-bacterial Bioinformatics Database and Analysis Resource Center. Nucleic Acids Res 45:D535–D542.

23.     R: The R Project for Statistical Computing.

24.     Wickham H, Averick M, Bryan J, Chang W, McGowan L, François R, Grolemund G, Hayes A, Henry L, Hester J, Kuhn M, Pedersen T, Miller E, Bache S, Müller K, Ooms J, Robinson D, Seidel D, Spinu V, Takahashi K, Vaughan D, Wilke C, Woo K, Yutani H. 2019. Welcome to the Tidyverse. Journal of Open Source Software 4:1686.

25.     Wilke CO. 2019. cowplot: Streamlined Plot Theme and Plot Annotations for “ggplot2.”

26.     Yu G, Smith DK, Zhu H, Guan Y, Lam TT-Y. 2017. ggtree: an r package for visualization and annotation of phylogenetic trees with their covariates and other associated data. Methods in Ecology and Evolution 8:28–36.

27.     Yu G, Lam TT-Y, Zhu H, Guan Y. 2018. Two Methods for Mapping and Visualizing Associated Data on Phylogeny Using Ggtree. Mol Biol Evol 35:3041–3043.

28.     Paradis E, Schliep K. 2019. ape 5.0: an environment for modern phylogenetics and evolutionary analyses in R. Bioinformatics 35:526–528.

29.     grid package | R Documentation.

30.     Wyres KL, Lam MMC, Holt KE. 2020. Population genomics of Klebsiella pneumoniae. Nature Reviews Microbiology 1–16.
